# The relevance of RT–PCR markers for metastatic tumour cell detection

**DOI:** 10.1038/sj.bjc.6603131

**Published:** 2006-05-02

**Authors:** M Chechlińska, M Kowalewska, S Markowicz, R Nowak

**Affiliations:** 1 Department of Immunology, The Maria Skłodowska-Curie Memorial Cancer Centre and Institute of Oncology, Roentgena 5, 02-781 Warsaw, Poland; 2 Department of Molecular Biology, The Maria Skłodowska-Curie Memorial Cancer Centre and Institute of Oncology, Roentgena 5, 02-781 Warsaw, Poland; 3 Postgraduate School of Molecular Medicine, Warsaw, Poland

**Sir**,

In a recently published paper in your journal Nissan *et al* (2006), following the commonly used definition, described ‘a molecule expressed in all tumour cells but not in normal human tissues’ as an ideal marker for minimal residual disease detection. In numerous studies based on this definition, the expression of markers thought to characterise disseminated cancer cells in the peripheral blood, bone marrow or nodules is analysed against the results obtained in the relevant tissue obtained from healthy donors. We raise an issue that healthy donor tissues are not adequate controls.

The same volume of BJC publishes a paper (one of a series) on the association of cancer patients’ outcomes with the inflammation-based prognostic score (Crumley *et al*, 2006). This and many other studies clearly indicate the presence and importance of a systemic inflammation in cancer patients, implying the very likely presence of activated lymphoid cells in blood, bone marrow or nodules. Yet, this phenomenon is evidently ignored in many studies on disseminated cancer cell RT–PCR detection. As we have shown, many of the so-called tumour markers (squamous-cell carcinoma antigen, epidermal growth factor receptor, mammaglobin and small breast epithelial mucin) are expressed in normal peripheral blood lymphocytes following polyclonal activation *in vitro* (unpublished data). Similarly, other researchers have demonstrated that some marker expression is inducible by cytokines in lymphoid cells from patients without epithelial cancer (Jung *et al*, 1998, Krüger *et al*, 2001). Therefore, a positive RT–PCR result may not necessarily indicate the presence of a cancer cell, but of an activated, inflammatory cell instead. As a result, RT–PCR detection, characterised by high sensitivity, shows low specificity. Consequently, the last decade's constant attempts to apply RT–PCR for micrometastasis detection have so far failed to produce a commonly accepted, routinely applied diagnostic method.

The proper assessment of new tumour markers should consider the inflammation-induced expression. The marker expression in nonmalignant inflammatory diseases would be informative and this is an interesting issue for basic science studies, but it represents an unrealistic approach while validating new molecular markers for metastatic cancer cell detection, as each new marker would require studies of peripheral blood, bone marrow, and nodules of numerous untreated patients. Instead, a simple model of lymphoid cell activation, such as *in vitro* mitogen-stimulated normal peripheral blood cells could serve for testing the specificity of new markers.
